# Nuclear Genome-Encoded Long Noncoding RNAs and Mitochondrial Damage in Diabetic Retinopathy

**DOI:** 10.3390/cells10123271

**Published:** 2021-11-23

**Authors:** Ghulam Mohammad, Renu A. Kowluru

**Affiliations:** 1 Department of Ophthalmology, Visual & Anatomical Sciences, Wayne State University, Detroit, MI 48201, USA; ed5563@wayne.edu; 2 Kresge Eye Institute, 4717 St. Antoine, Detroit, MI 48201, USA

**Keywords:** diabetic retinopathy, long noncoding RNA, mitochondria

## Abstract

Retinal mitochondria are damaged in diabetes-accelerating apoptosis of capillary cells, and ultimately, leading to degenerative capillaries. Diabetes also upregulates many long noncoding RNAs (LncRNAs), including Lnc*MALAT1* and Lnc*NEAT1*. These RNAs have more than 200 nucleotides and no open reading frame for translation. Lnc*MALAT1* and Lnc*NEAT1* are encoded by nuclear genome, but nuclear-encoded LncRNAs can also translocate in the mitochondria. Our aim was to investigate the role of Lnc*MALAT1* and Lnc*NEAT1* in mitochondrial homeostasis. Using human retinal endothelial cells, the effect of high glucose on Lnc*MALAT1* and Lnc*NEAT1* mitochondrial localization was examined by RNA fluorescence in situ hybridization. The role of these LncRNAs in mitochondrial membrane potential (by JC-I staining), mtDNA integrity (by extended length PCR) and in protective mtDNA nucleoids (by SYBR green staining) was examined in *MALAT1-* or *NEAT1-siRNA* transfected cells. High glucose increased Lnc*MALAT1* and Lnc*NEAT1* mitochondrial expression, and *MALAT1*-siRNA or *NEAT1*-siRNA ameliorated glucose-induced damage to mitochondrial membrane potential and mtDNA, and prevented decrease in mtDNA nucleoids. Thus, increased mitochondrial translocation of Lnc*MALAT1* or Lnc*NEAT1* in a hyperglycemic milieu plays a major role in damaging the mitochondrial structural and genomic integrity. Regulation of these LncRNAs can protect mitochondrial homeostasis, and ameliorate formation of degenerative capillaries in diabetic retinopathy.

## 1. Introduction

Diabetes is one of the fastest growing health challenges of the 21st century, and ~80% of patients develop retinopathy after 15 years of diabetes; worldwide, 17 million people have proliferative diabetic retinopathy. In the pathogenesis of this sight-threatening disease, mitochondrial dysfunction plays a major role; mitochondrial structural, functional and genomic stability in the retina and its vasculature is impaired, and the damaged mitochondria fuel into the vicious cycle of free radicals, accelerating capillary cell apoptosis, a phenomenon which precedes the development of diabetic retinopathy [1,2,3].

Evolving research has shown that only one-fifth of the transcription across the human genome is associated with protein-coding genes, and the non-protein-coding portion of the human genome is ~4 times more than the coding RNA sequences. Among these noncoding RNAs, long noncoding RNAs (LncRNAs) are noncoding transcripts longer than 200 nucleotides that can bind to DNA or RNA in a sequence-specific manner, bind to proteins, or can act as sponges for miRNAs [4,5,6]. LncRNAs are a large and heterogeneous group of functional RNAs that are mainly encoded by nuclear DNA, and are shown to play crucial roles in the regulation of pathophysiological processes in many chronic diseases including cancer, diabetes and its complications [7,8,9,10,11]. In diabetic retinopathy, expression of many LncRNAs, including metastasis-associated lung adenocarcinoma transcript 1 (*MALAT1*), antisense noncoding RNA in the *INK4* locus (*ANRIL*), nuclear-enriched abundant transcript 1 (*NEAT1*), brain-derived neurotrophic factor antisense (*BDNF****-****AS*) and HOXA distal transcript antisense RNA (*HOTTIP*) are upregulated, contributing to an increase in inflammatory markers, VEGF, angiogenesis, apoptosis and oxidative stress [11,12,13,14].

Two of the highly expressed LncRNAs that are also implicated in apoptosis and autophagy, Lnc*MALAT1* and Lnc*NEAT1*, are encoded by nuclear genome, and then distributed in the cytoplasm to associate with diverse RNA-binding proteins [15]. However, a recent study has shown that Lnc*MALAT1* interacts with multiple loci on the mitochondrial DNA (mtDNA) in hepatoma cells and regulates mitochondrial homeostasis [16]. Lnc*NEAT1*, which is intimately associated with paraspeckles formation, is rich in the nucleus, but, it is also found in subcellular space [17], and depletion of Lnc*NEAT1* is shown to affect mitochondrial dynamics and function by altering the sequestration of nuclear-encoded mitochondrial proteins in paraspeckles [18]. However, the role of these LncRNAs in mitochondrial homeostasis in diabetic retinopathy remains unclear.

The aim of this study was to investigate the role of nuclear genome-encoded LncRNAs in mitochondrial homeostasis in diabetic retinopathy. Using human retinal endothelial cells, mitochondrial localization of Lnc*MALAT1* and Lnc*NEAT1* in high glucose was examined. The role of these LncRNAs in mitochondrial structural and genomic stability was investigated in the cells transfected with their respective siRNAs.

## 2. Methods

Human retinal endothelial cells (HRECs, Cat. No. ACBRI 181, Cell Systems Corp., Kirkland, WA, USA) were cultured in Dulbecco’s Modified Eagle Medium (Cat No. D5523, Sigma-Aldrich, St. Louis, MO, USA) containing 12% heat-inactivated fetal bovine serum, 15 μg/mL endothelial cell growth supplement and 1% each insulin, transferrin, selenium, glutamax and antibiotic/antimitotic. Cells from the 5th–8th passage were incubated in 5 mM or 20 mM d-glucose for 96 h (NG and HG, respectively). As an osmotic/metabolic control, parallel incubations were run where cells were incubated in 20 mM l-glucose, instead of 20 mM d-glucose [19,20].

Using Lipofectamine RNAiMAX transfection reagent (Cat. No. 13778-030, Invitrogen™, Carlsbad, CA, USA), cells from the 5th–6th passage were transfected with Silencer^TM^ Select *MALAT1*-siRNA and or *NEAT1*-siRNA (Cat. No. 4392420, n272231 and Cat. No. 4390771, n272455, respectively, Ambion, Carlsbad, CA, USA). The transcription complex, prepared by adding 0.25–1µg siRNA duplex to the transfection reagent, was incubated at 37 °C for 30 min. This was followed by incubating HRECs (pre-rinsed with the transfection medium, Opti-MEM) in the transfection complex for 8 h at 37 °C. At the end of the incubation, the cells were incubated in 5 mM or 20 mM d-glucose for 96 h [11]. As a control, each transfection experiment included cells transfected with a non-targeting scrambled RNA. Each experiment had HRECs from the same batch and the same passage, and was repeated 3–4 times [11]. The transfection efficiency, evaluated by quantifying the gene transcripts of the respective genes, was ~50%.

### 2.1. RNA Sequencing

Nuclear fraction was isolated by centrifugation using a kit from Thermo-Fisher Scientific (Cat. No. 89874, Waltham, MA, USA) from HRECs incubated in normal and high d-glucose, or l-glucose, and was outsourced to GENEWIZ (South Plainfield, NJ, USA) for library preparation and Illumina next-generation sequencing. The raw FASTQ sequencing files were uploaded in the www.usegalaxy.org (accessed on 21 May 2021) server and aligned against the hg38 human genome dataset using the TopHat module. Fold change and fragments per kilobase of exon per million reads (FPKM) were calculated from the aligned bam files using stringTie modules [11].

### 2.2. Gene Expression

RNA was isolated by Trizol (Cat. No. 15596018, Ambion, Carlsbad, CA, USA), and converted to cDNA using the High-Capacity cDNA Reverse Transcription Kit (Applied Biosystems, Foster City, CA, USA). Gene transcripts were quantified by SYBR green-based real-time quantitative PCR (qRT-PCR) using gene-specific primers (Table 1) and β-actin as the housekeeping gene [19,20].

### 2.3. RNA Fluorescence In Situ Hybridization (RNA-FISH)

Using asymmetric PCR amplification, fluorescein-12-dUTP incorporated probe was prepared, and the PCR products were gel-purified, as described recently [11]. HRECs were fixed with 4% paraformaldehyde, followed by their dehydration using 70% to 100% ethanol. They were air-dried and incubated at 37 °C for two hours with denatured probe in formamide-containing buffer. After washing with the hybridization buffer, the cells were then washed with PBS and mitochondrial localization was performed by immunofluorescence technique using antibody for the mitochondrial marker CoxIV (Cat No: ab33985; Abcam, 1:100 dilution). The secondary antibody, Texas red-labeled anti-mouse, was used at 1:100 dilution. The coverslips were mounted using Vectashield mounting medium (Cat. No. H-1000, Vector Laboratories, Burlingame, CA, USA), and images were captured by Zeiss microscope at 40 × objective using the Apotome module. Signals of fluorescein-12-dUTP incorporated probe were visualized to determine the hybridized probes. The captured images were calibrated with the ZEISS proinbuilt software package and modules. Using the Zeiss colocalization software module, the Pearson’s correlation coefficient between CoxIV and Lnc*MALAT1* or Lnc*NEAT1* was calculated using a “Region of Interest”, which allowed us to exclude the image background. “Region of Interest” was created manually by circle selection tool using the Zeiss software module, and Pearson’s correlation coefficient within the “Region of Interest” was calculated by keeping the same area.

### 2.4. Reactive Oxygen Species

Mitochondrial ROS levels were also quantified by using a mitochondrial superoxide indicator MitoSox. Live cells were incubated with 5 μM MitoSox red (Cat No. M36008, Thermo Fisher) and 200 nM MitoTracker Green for 20 min at 37 °C. The cells were then imaged under a Zeiss ApoTome at 40× objective [20]. The intensity was quantified by Zeiss software module (Carl Zeiss, Inc., Chicago, IL, USA).

### 2.5. Mitochondrial Membrane Potential

Mitochondrial membrane potential changes (ΔΨm) were measured by staining cells with a mitochondrial binding dye, JC-1 (Cat. No. MP03168, Molecular Probes, Carlsbad, CA, USA) as described previously [20]. Briefly, after experimental incubations, cells were washed with PBS and incubated with DMEM containing 5 µM JC-1 for 30 min at 37 °C. Cells were then again washed with PBS, and visualized under Zeiss ApoTome fluorescence microscope at 20× objective. The fluorescence intensity of the monomers (green, at 485 nm excitation and 530 nm) and the aggregates (red, at 525 nm excitation and 590 nm emission) was determined using Zeiss software module, and the ratio of red to green intensity was calculated as ΔΨm [21,22].

### 2.6. Mitochondrial DNA Damage

Exploiting the ability of the damaged DNA to prevent polymerase progression along the DNA template, extended length PCR was performed [23,24] using GeneAmp XL PCR kit (Applied Biosystems). In brief, long and short mtDNA regions (8.8 kbp and 117 bp, respectively) were amplified by semiquantitative PCR. The amplified products were separated on 1.2% and 2.0% agarose gel for long and short amplification. Relative amplification was quantified by normalizing the intensity of the long product to the short product; the degree of the damage was inversely proportional to the ratio [20,25,26].

### 2.7. Mitochondrial Nucleoids

HRECs plated on coverslips and incubated in high glucose medium were fixed with 100% methanol for 15 min. The cells were permeabilized with 0.5% tritonX100, washed with PBS, and incubated in 1 mL 1× SYBR Green stain S333102, Invitrogen) for 1 h at room temperature [27]. After washing the cells with PBS (5 min × 2), they were mounted using Vectashield plus antifade mounting medium for imaging. Images were acquired using 40× objective lens on a ZEISS microscope with excitation of a 488 nm wavelength filter. The images were analyzed using the ZEISS software module and the nucleoid foci were counted with automated counting protocol, by adjusting the threshold and keeping a size factor of 14 as selection criteria for the smallest foci in the ZEISS analysis module. To further confirm nucleoids in the mtDNA, the co-immunofluorescence technique was performed using SYBR green and mtDNA-encoded cytochrome b (Cytb) as a mitochondrial marker. For Cytb, the primary antibody was used at 1:1000 dilution (Cat. No. SAB4301200, Sigma-Aldrich), and the secondary antibody was Texas-Red-conjugated anti-rabbit (Cat. No. TI-1000, Vector Laboratories, Burlingame, CA, USA, 1:500 dilution) [28]. The coverslips were mounted using DAPI-containing mounting medium (Vector Laboratories), and images were captured by Zeiss microscope at 40× objective using the Apotome module. The intensity profile was determined by “line region of interest”, and Pearson’s coefficient was evaluated using Zeiss software module.

### 2.8. Statistical Analysis 

Statistical analysis was performed using Graph Pad Prism (San Diego, CA, USA). The data are presented as mean ± SD, and group comparisons were performed using one-way ANOVA followed by Dunn’s *t*-test. A *p* < 0.05 was considered significant.

## 3. Results

**Figure 1:**
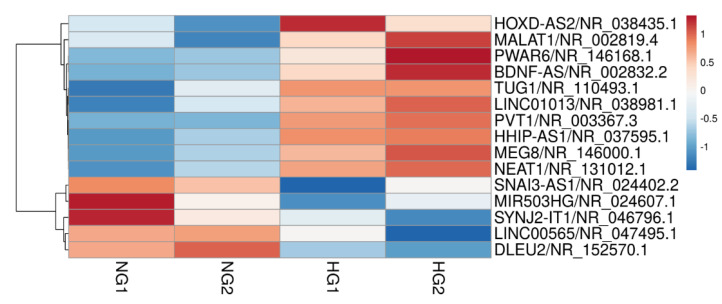
Heatmap analysis of LncRNAs in HRECs incubated in high glucose. RNA sequencing was performed in the nuclear fraction of the cells incubated in normal and high glucose; the figure represents a heatmap of two different cell preparations, each incubated in 5 mM d-glucose (NG1 and NG2) and in 20 mM d-glucose (HG1 and HG2).

The sequencing data showed increased expression of many LncRNAs in the cells in high glucose, compared to cells in normal glucose. Similarly, several LncRNAs were also downregulated in high glucose; the representative heatmap shows upregulated and downregulated LncRNAs (Figure 1).

**Figure 2:**
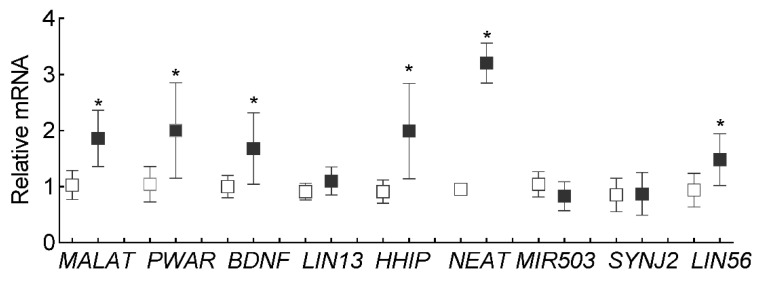
Validation of differently expressed LncRNAs in high glucose. Nine differentially expressed LncRNAs from the heatmap were quantified by qRT-PCR using β-actin as the housekeeping gene. Each measurement was made in duplicate in three different cell preparations. Values obtained from cells in NG were considered as 1, and are represented as mean ± SD. * *p* < 0.05 vs. NG.

To confirm the results obtained from the Heatmap, qRT-PCR was performed. Consistent with our previous results [11], Lnc*MALAT1* expression was upregulated by ~2 fold in cells incubated in high glucose compared to cells in normal glucose. In addition to Lnc*MALAT1*, Lnc*PWAR6*, Lnc*BDNF1AS* and Lnc*HHIP-AS1* were also upregulated by ~2 fold, and Lnc*NEAT1* by >3 fold (Figure 2).

**Figure 3:**
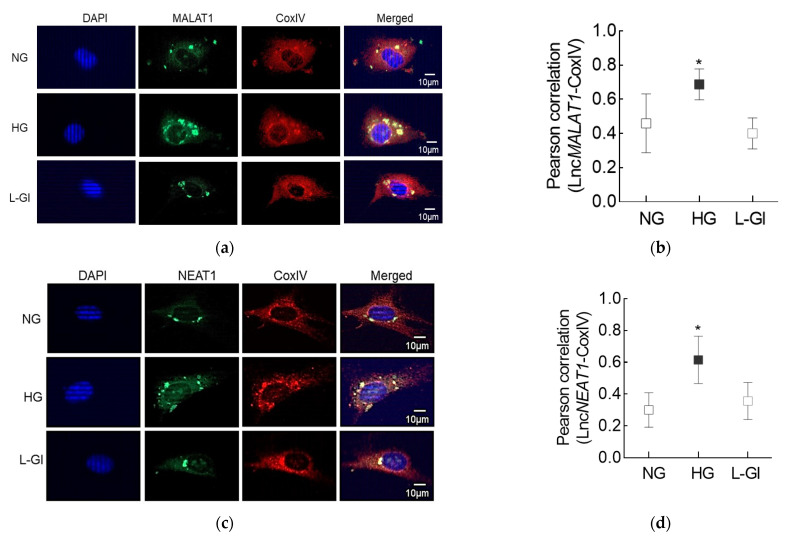
Effect of high glucose on mitochondrial localization of Lnc*MALAT1* and Lnc*NEAT1*. (**a**,**c**) Using RNA-FISH technique, cells fixed with 4% paraformaldehyde were incubated with fluorescein-labelled denatured probes. Mitochondrial localization was performed using CoxIV antibody and Texas Red-conjugated secondary antibody. Cells were mounted using DAPI (blue)-containing mounting medium. (**b**,**d**) Signals of fluorescein-12-dUTP incorporated probe (green) were visualized to determine the hybridized probes. Pearson’s correlation coefficient of CoxIV with Lnc*MALAT1* or Lnc*NEAT1* was calculated by using the “Region of Interest” in the outer nuclear region from 25 or more cells in each group. Pearson’s correlation coefficient values in the graphs are represented as mean ± SD. NG and HG = Cells in 5 mM or 20 mM d-glucose; L-Gl = cells in 20 mM l-glucose; * *p* < 0.05 vs. NG.

Lnc*MALAT1* and Lnc*NEAT1*, the two highly expressed LncRNAs, are also implicated in mitochondrial homeostasis [16,18], to understand the role of these nuclear genome-encoded LncRNAs in mitochondrial dysfunction in diabetic retinopathy; the proceeding experiments were focused on Lnc*MALAT1* and Lnc*NEAT1.* RNA-FISH analysis showed significantly increased fluorescence intensity for both Lnc*MALAT1* and Lnc*NEAT1* in the mitochondria of the cells incubated in high glucose compared to cells in normal glucose. However, cells incubated in 20 mM l-glucose had similar mitochondrial localization of Lnc*MALAT1* or Lnc*NEAT1* as cells in normal glucose. Quantification of the Pearson’s correlation coefficient of CoxIV and Lnc*MALAT1* or Lnc*NEAT1* from 20–30 cells in each group showed significantly higher Pearson’s correlation coefficient of CoxIV with Lnc*MALAT1* or Lnc*NEAT1* in the cells in high glucose vs. cells in normal glucose (Figure 3a–d).

**Figure 4:**
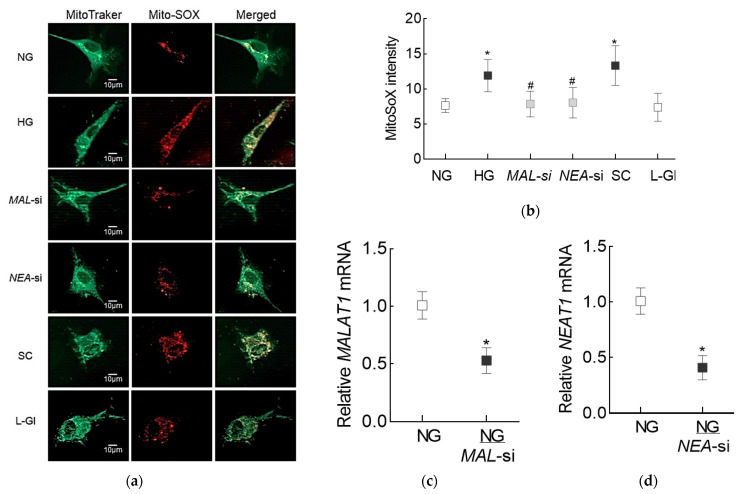
Effect of inhibition of Lnc*MALAT1* or Lnc*NEAT1* on high glucose-induced mitochondrial ROS levels. (**a**) Live cells, incubated with MitoSox red and MitoTracker green, were imaged under a Zeiss Apotome at 40× objective. (**b**) Mean MitoSox intensity, obtained from 30–50 cells, in each incubation condition. (**c**,**d**) Transfection efficiency of *MALAT1* and *NEAT1*, as determined by their respective gene transcripts. Each measurement was made in duplicate in 2–3 different cell preparations. NG and HG = Cells in 5 mM or 20 mM d-glucose, *MAL*-si and *NEAT*-si = Cells transfected with *MALAT1*-siRNA or *NEAT1*-siRNA and incubated in high glucose, SC = Cells transfected with scrambled control RNA and incubated in high glucose, NG/*MAL*-si and NG/*NEA*-si = cells transfected with *MALAT1*-siRNA or *NEAT1*-siRNA in normal glucose, and L-Gl = Cells incubated in 20 mM l-glucose. * *p* < 0.05 vs. NG and # *p* < 0.05 vs. HG.

To determine the role of these two LncRNAs in modulating glucose-induced oxidative stress, mitochondrial ROS were measured in the cells transfected with *MALAT1*-siRNA or *NEAT1*-siRNA, and incubated in high glucose. As reported previously [29], mitochondrial ROS levels in cells in high glucose were increased, compared to cells in normal glucose. Regulation of LncRNAs by their respective siRNAs prevented glucose-induced increase in mitochondrial ROS; the values obtained from the transfected cells in high glucose were not different from the un-transfected cells in normal glucose. However, cells transfected with scrambled control RNA were not protected from glucose-induced increase in mitochondrial ROS, and the values were significantly higher compared to cells transfected with either *MALAT1*-siRNA or *NEAT1*-siRNA (Figure 4a,b). Figure 4c,d represents the transfection efficiency of *MALAT1*-siRNA and *NEAT1*-siRNA, respectively.

**Figure 5:**
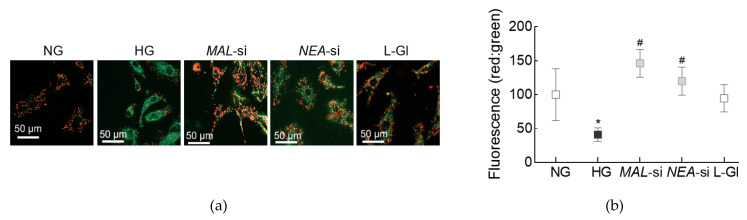
Silencing of *MALT1* or *NEAT1* on mitochondrial membrane integrity. (**a**) Representative image obtained using cationic dye JC-1 to determine mitochondrial membrane potential. Cells were visualized under a Zeiss ApoTome fluorescence microscope at 20× objective. Green fluorescence represents the depolarized (monomer) mitochondria and orange represents hyperpolarized (J aggregates) mitochondria. (**b**) The graph represents the ratio of intensity of red and green fluorescence. * *p* < 0.05 vs. NG and # *p* < 0.05 vs. HG.

The role of Lnc*MALAT1* and Lnc*NEAT1* in mitochondrial damage was determined by measuring mitochondrial membrane potential in the cells transfected with *MALAT1*-siRNA or *NEAT1*-siRNA. As shown in Figure 5, cells in normal glucose had significantly higher red fluorescent J-aggregates, compared to cells in high glucose. However, compared to un-transfected cells, or cells transfected with scrambled non-targeting control RNA, in high glucose, red fluorescent J-aggregates were higher in *MALAT1*-siRNA or *NEAT1*-siRNA transfected cells. The accompanying graph shows the fluorescence intensity of red/green in each of the incubation conditions. Incubation of un-transfected cells in 20 mM l-glucose had no effect on mitochondrial membrane potential, and the red fluorescent aggregates were similar to those obtained from cells in normal glucose.

**Figure 6:**
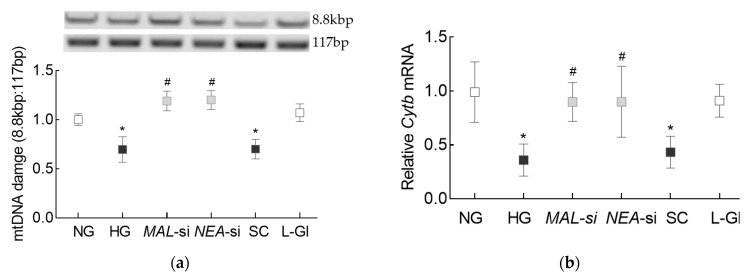
Mitochondrial DNA damage and or Lnc*NEAT1*. (**a**) Mitochondria DNA damage was quantified by extended-length PCR using long mtDNA (8.8 kbp) and short (117 bp) amplicons of the mtDNA. (**b**) *Cytb* gene transcripts were quantified by qRT-PCR using β-actin as the housekeeping gene. Each measurement was made in duplicate/triplicate in 3–4 different cell preparations, and the histograms represent values as mean ± SD. NG = 5 mM d-glucose; HG = 20 mM d-glucose, *MAL*-si, *NEAT*-si and SC = Cells transfected with *MALAT1*-siRNA, *NEAT1*-siRNA, or scrambled control RNA respectively, and incubated in 20 mM d-glucose, L-Gl = cells in 20 mM l-glucose. * and # *p* < 0.05 vs. NG and HG respectively.

To investigate the effect regulation of these LncRNAs on mtDNA integrity, mtDNA damage was determined in *MALAT1*-siRNA or *NEAT1*-siRNA transfected cells. As shown previously [25], high glucose decreased the ratio of amplification of long (8.8 kbp) and short primer (117 bp) significantly, compared to cells in normal glucose, suggesting increased mtDNA damage. However, while cells transfected with scrambled non-targeting RNA and un-transfected cells in high glucose had similar ratios, cells transfected with *MALAT1*-siRNA or *NEAT1*-siRNA had significantly higher ratios, implying less damage to the mtDNA (Figure 6a). Consistent with protection of mtDNA damage, *NEAT1*-siRNA also protected glucose-induced decrease in mtDNA transcription; compared to un-transfected cells in high glucose, gene transcripts of mtDNA-encoded *Cytb* of complex III of the electron transport chain system were significantly higher in cells transfected with either *MALAT1*-siRNA or *NEAT1*-siRNA (Figure 6b).

**Figure 7:**
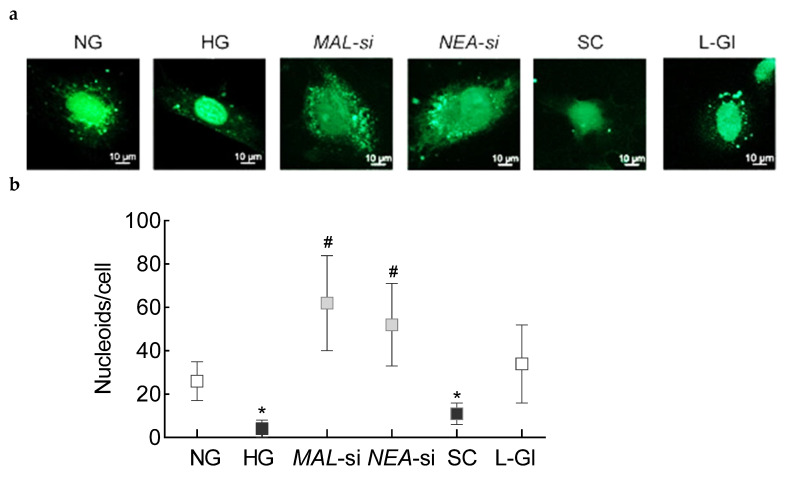

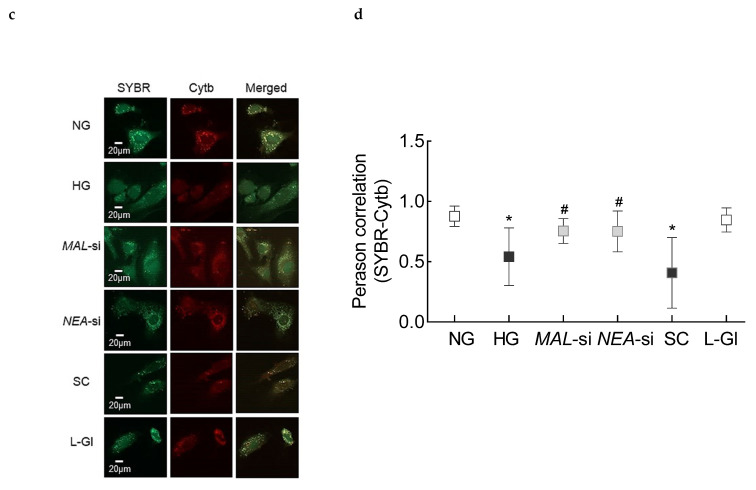
Effect of silencing of *MALAT1* or *NEAT1* on nucleoids in mtDNA: (**a**) Nucleoids were quantified by staining cells using SYBR (green) and imaging at 40× objective. (**b**) The histogram represents the number of nucleoids from 5 to 8 images/group, quantified using the ZEISS analysis module. (**c**) Representative image of the localization of nucleoids in the mitochondria using SYBR (green) and mitochondrial marker Cytb (Cytb, red) staining. (**d**) The images were calibrated with the ZEISS proinbuilt software package, and using random region of interest in the area outside the nucleus, Pearson’s correlation coefficient between red and green stain was determined. Each measurement was made in duplicate in 3–4 different cell preparations. NG = 5 mM d-glucose; HG = 20 mM d-glucose, *MAL*-si, *NEAT*-si and SC = Cells transfected with *MALAT1*-siRNA, *NEAT1*-siRNA, or scrambled control RNA, respectively, and incubated in 20 mM d-glucose, L-Gl = cells in 20 mM l-glucose. * *p* < 0.05 vs. NG and # *p* < 0.05 vs. HG.

Mitochondrial DNA has nucleoprotein complexes composed of numerous nucleoid-associated proteins to provide a stable environment for mtDNA replication and repair [30]. The effect of regulation of Lnc*MALAT1* and Lnc*NEAT1* on mtDNA nucleoids was investigated. Nucleoids were decreased significantly in HRECs in high glucose, compared to cells in normal glucose, and transfection with *MALAT1*-siRNA or *NEAT1*-siRNA, but not scrambled RNA, prevented glucose-induced decrease in nucleoids (Figure 7a). To further confirm the localization of the nucleoids in the mtDNA, Figure 7b shows decreased Pearson’s correlation co-efficient between SYBR green and mtDNA-encoded Cytb in cells in high glucose vs. cells in normal glucose, but similar correlation co-efficient in *MALAT1*-siRNA or *NEAT1*-siRNA transfected cells in high glucose and un-transfected cells in normal glucose.

## 4. Discussion

Multiple pathways are implicated in the development of diabetic retinopathy, making the pathogenesis of this blinding disease very complex [1,31]. Mitochondrial dysfunction is considered to play an important role in the development of diabetic complications, including retinopathy; diabetic environment increases oxidative stress in the retina and its vasculature, which dysfunctions the mitochondria. Dysfunctional mitochondria accelerate apoptosis of capillary cells, leading to the formation of degenerative capillaries and pericyte ghosts in the retinal vasculature, and poor or no perfusion of these acellular capillaries subsequently results in neovascularization [1,2,32,33]. In addition to structural and functional damage to the mitochondria, expression of many genes associated with mitochondrial homeostasis is also altered [25,34,35]. Regulation of almost every stage of gene expression is also mediated by LncRNAs, the noncoding RNAs with over 200 nucleotides and higher tissue specificity compared to miRNAs [36]. Although most of the LncRNAs function outside the mitochondria, several nuclear genome-encoded LncRNAs are also found in the mitochondria, and are implicated in mitochondrial function and dynamics [37,38,39]. This is the first report demonstrating the importance of nuclear genome-encoded Lnc*MALAT1* and Lnc*NEAT1* in the mitochondrial homeostasis in diabetic retinopathy. We show that high glucose increases the expression of both of these LncRNAs in the mitochondria in retinal endothelial cells, and regulation of these two LncRNAs by their specific siRNAs, ameliorates glucose-induced increase in oxidative stress and mitochondrial structural and genomic damage.

Diabetic patients have increased circulating expressions of several LncRNAs including Lnc*MALAT1* and Lnc*NEAT1* [40]. Lnc*MALAT1* is one of the most extensively studied and highly conserved LncRNA, and is expressed at relatively high level in almost all human tissues. Lnc*MALAT1* is implicated in normal physiologic functions such as vascular growth, and in pathophysiological processes in many diseases including vascular and neurologic disorders and cancers [41]. In diabetes, its expression is elevated in various tissues, and emerging evidence has shown that Lnc*MALAT1* acts as both pro-inflammatory and apoptotic [42,43,44]. Increased Lnc*MALAT1* is seen in the retina, aqueous humor and fibrovascular membranes from patients with diabetes [11,45]. Regulation of Lnc*MALAT1* prevents cell migration and angiogenesis in retinal endothelial cells, alleviates neurodegeneration and inhibits monocyte chemotactic protein-1, and ameliorates inactivation of the master transcription factor, Nuclear factor erythroid 2-related factor, Nrf2, in rodent models of diabetic retinopathy [11,46,47]. Similarly, Lnc*NEAT1* is shown to act as an important sensor and effector during stress and disease development [48,49]. Our data show that high glucose elevates the levels of both of these LncRNAs in HRECs, compared to normal glucose.

Genes for LncRNAs, compared to miRNAs, are less evolutionarily conserved and less abundantly expressed [50]. Other than three LncRNAs encoded by mitochondrial genome, LncRNAs are encoded by nuclear genome, exported to the cytosol where they are distributed in the cytoplasm and associate with diverse RNA-binding proteins [15]. However, recent evidence has suggested that mammalian mitochondria can also import LncRNAs from the cytosol [38]. Here, our results clearly show that both Lnc*MALAT1* and Lnc*NEAT1* are transported in the mitochondria, and their mitochondrial accumulation increases significantly in hyperglycemic milieu. In support, increased translocation of Lnc*MALAT1* from the nucleus to the mitochondria is seen in hepatocellular carcinoma cells [16,39]. 

LncRNAs in the mitochondria, whether encoded by the mitochondrial genome or nuclear genome and then transported into the mitochondria, play an essential role in mitochondrial homeostasis; *MALAT1* knockout mice have reduced ROS and ROS-generated protein carbonylation in hepatocyte and islets [51], and Lnc*NEAT1*, via downregulating miR-128 in macrophages, is shown to reduce inflammation and oxidative stress [49]. Reduced mitochondrial membrane potential is considered as an initial and irreversible step towards apoptosis as it redistributes cytochrome c from the cristae to the intermembrane space, making it more susceptible to release [52]. In the pathogenesis of diabetic retinopathy, mitochondrial membrane is damaged and membrane potential is decreased, facilitating the release of cytochrome c from mitochondria into the cytosol, and initiating the apoptotic process [1,2,3]. Our results clearly show that an increase in ROS levels and damage to the mitochondrial membrane integrity by preventing an increase in membrane potential, that the cells experience in high glucose, are ameliorated by specific siRNAs of Lnc*MALAT1* or Lnc*NEAT1*. The results presented here showing the role of nuclear genome-encoded LncRNAs regulating mitochondrial functions are supported by reports showing regulation of mitochondrial functions in podocytes in diabetic nephropathy by nuclear genome-encoded LncRNA taurine-upregulated gene 1 [53], and aberrant NEAT1 expression by mitochondrial stressors [18].

Retinal mtDNA is damaged in diabetic retinopathy, and transcription of mtDNA-encoded genes, important for the functioning of the electron transport chain, is impaired, resulting in a vicious cycle of free radicals [1,2,3]. Data presented here demonstrate that glucose-induced mtDNA damage is prevented by the siRNAs of Lnc*MALAT1* or Lnc*NEAT1*. Others have shown that *MALAT1* interacts with multiple loci on mtDNA in hepatoma cells [16], and NEAT1 plays a role in DNA repair processes [54]. Mitochondrial DNA lacks protective histones, but it has nucleoprotein complexes, the nucleoids, that provide a stable environment for mtDNA replication and repair [30]. Nucleoids also constitute a switch for controlling mitochondrial metabolism in response to cellular demands [55,56]. The results show that high glucose decreases mtDNA nucleoids in retinal endothelial cells, and Lnc*MALAT1* or Lnc*NEAT1* siRNA protects mtDNA from loss of nucleoids, suggesting that increased expressions of these LncRNAs in hyperglycemic conditions make the histone-free mtDNA more vulnerable to the damage. In support, LncRNAs are also shown to affect the interaction of proteins altering the genomic stability [57,58,59].

We recognize that this study is focused on Lnc*MALAT1* or Lnc*NEAT1*, the two LncRNAs that are highly expressed, but there are many other LncRNAs that are altered in diabetic retinopathy, and the possibility that other LncRNAs could be altering mitochondrial homeostasis in the development of diabetic retinopathy cannot be ruled out. Also, how hyperglycemic milieu facilitate their transport inside the mitochondria remains to be explored. Our main focus was on the role of these LncRNAs in mitochondrial homeostasis, and to acknowledge the roles of Lnc*MALAT1* or Lnc*NEAT1* in regulating other important metabolic abnormalities that are implicated in the development of diabetic retinopathy.

In summary, hyperglycemia enables nuclear genome-encoded Lnc*MALAT1* and Lnc*NEAT1* to transport inside the mitochondria, increasing ROS and damaging their membranes. Once inside the mitochondria, these LncRNAs damage mtDNA and reduce their protective nucleoids, and the transcription of mtDNA-encoded genes is impaired, increasing the vulnerability of the electron transport chain system. Thus, regulation of these LncRNAs could provide the already vulnerable mitochondria with some protection for initiating a futile, self-propagating cycle of free radicals in diabetic retinopathy.

## Figures and Tables

**Table 1:** Primer sequences.

Gene	Sequence
*MALAT1*	Fwd-GCC ATT CCA GGT GGT GGT ATT TAG Rev-GCA GAT TCT GTG TTA TGC CTG GTT AG
*PWAR*	Fwd-GGG CAT AGC AGT GGA AAC TCA AA Rev-CAA GCA CAG TAT AAC AAG GCC AGA G
*BDNF*	Fwd-CAG CAA CCG CGA CTA CCA AAT A Rev-GAA GGG ATT CTG TTG GGT GCT AAA
*LIN13*	Fwd-AGT CTG AGG AGG CAG TTT CCT ATC Rev-CTG GAT GCC AAC TAG CAC AGT TT
*HHIP*	Fwd-GGA GGC TGA AGA AGC AGA GGA TAG Rev-GCT GGA AAG GGC CGA TTT GAT T
*NEAT1*	Fwd-CCT GCC TTC TTG TGC GTT TCT Rev-ACT TGT ACC CTC CCA GCG TTT A
*MIR503*	Fwd-AAC CAC CCA AGT GTC CCA AAT AG Rev-TGG AAC AAA GAA GTG TGG GTA TGG
*SYNJ2*	Fwd-TCT GGC CTC CAG GAT GAC TAT TT Rev-TGC CTG TAA TCC CAG CAC TTT G
*LIN56*	Fwd-ACT GGC CTG AGC ATT GCA TAA C Rev-TTG GAT GGA CCA ACG TGC TTT C
*β-Actin*	Fwd-AGC CTC GCC TTT GCC GAT CCG Rev-TCT CTT GCT CTG GGC CTC GTCG
*mtDNA short*	Fwd-CCT CCC ATT CAT TAT CGC CGC CCT TGC Rev-GTC TGG GTC TCC TAG TAG GTC TGG GAA
*mtDNA long*	Fwd-AAA ATC CCC GCA AAC AAT GAC CAC CCC Rev-GGC AAT TAA GAG TGG GAT GGA GCC AA
*Cytb*	Fwd-TCA CCA GAC GCC TCA ACC GC Rev-GCC TCG CCC GAT GTG TAG GA

## Data Availability

R.A.K. is the guarantor of this work and, as such, had full access to all the data in the study and takes responsibility for the integrity of the data and the accuracy of the data analysis.
